# The Impact of Career Education Participation on Academic Self-Efficacy: The Sequential Mediating Role of Career Adaptability and Learning Engagement

**DOI:** 10.3390/bs15020174

**Published:** 2025-02-07

**Authors:** Chunmin Pang, Ruiqin Xie, Nan Kang, Zhongfeng Hu

**Affiliations:** 1 School of Politics and Public Administration, South China Normal University, Guangzhou 510006, China; 2021010009@m.scnu.edu.cn; 2 Education Assessment Department, Guangdong Academy of Education, Guangzhou 510035, China; 3 Center for Education and Development of Nanhai District, Foshan 528299, China; xieruiqin@m.scnu.edu.cn; 4 School of Humanities and Social Science, Xi’an Jiaotong University, Xi’an 710049, China; kangnan@xjtu.edu.cn; 5 Center for Counseling and Psychological Development, Xi’an Jiaotong University, Xi’an 710049, China; 6 School of Education, Kashi University, Kashi 844000, China; 7 Research Center for Education and Social Integration in Guangdong-Hong Kong-Macao Greater Bay Area, South China Normal University, Guangzhou 510006, China; 8 School of Education (Shanwei), South China Normal University, Shanwei 516625, China

**Keywords:** career education participation, career adaptability, learning engagement, academic efficacy, sequential mechanism

## Abstract

In order to clarify the mechanism of how career education impacts academic self-efficacy among Chinese senior high school students, data from 848 senior high school students in China (*M_age_* = 16.88, *SD_age_* = 3.56) were obtained in this study. The goal was to test the independent and sequential mediating effects of career adaptability and learning engagement in the relationship between career education participation and academic self-efficacy. This study revealed that participation in career education significantly enhances academic self-efficacy. Specifically, career adaptability and learning engagement act as sequential mediators in the relationship between career education participation and academic self-efficacy. This study reveals one potential mechanism of how career education participation in schools impacts adolescent academic self-efficacy; the insights presented here have significant implications for the design of educational interventions.

## 1. Introduction

It is widely acknowledged that providing diverse career education opportunities for senior high school students is crucial for their future career success (Watts & Sultana, 2004). In 1994, *School-to-Work Opportunities* was enacted by the United States *Act* to drive nationwide reform in career education and to prepare teenagers for their future career (Granello & Sears, 1999). The United Kingdom, New Zealand, and numerous Scandinavian countries have also implemented career-planning programs with the objective of assisting young people in achieving success in their careers (Hutchinson, 2018; Yates & Bruce, 2017; Andreassen et al., 2019). Initiated in 2014, the latest round of reforms to China’s higher education entrance examination process has provided senior high school students with a broader range of academic options. Students are now afforded greater autonomy in choosing their examination subjects and university majors. In this context, the Chinese government has mandated the implementation of senior high school career education on a national scale, aiming to assist students in achieving their desired academic outcomes and facilitating a seamless transition from senior high school to university. In recent years, a significant proportion of China’s provinces have adopted a career education curriculum in senior high schools. This curriculum encompasses modules on career awareness, exploration, decision making, and management. It closely aligns with the career education framework outlined in *The National Development Guidelines* of the United States and *The Careers Framework (2018)* of the United Kingdom, and the implementation cycle is three years. As career education continues to be offered in senior high schools, questions have begun to emerge regarding its impact on students’ academic development, especially in relation to the psychological constructs that are associated with academic performance and the underlying mechanisms through which they occur. This study aimed to clarify the impact of the long-term systematic implementation of senior high school career education participation on students’ academic self-efficacy, with career adaptability and learning engagement serving as possible mediators. A total of 848 Chinese senior high school students participated in this study. Accordingly, this study and its findings contribute to a greater understanding of the above questions while addressing current research limitations.

Social cognitive theory (SCT) and its subsequent expansion, social cognitive career theory (SCCT), offer a comprehensive analytical framework that is particularly well-suited for the present study. Within the context of social cognitive theory and social cognitive career theory, self-efficacy is a fundamental construct in understanding individual career development. Individuals’ personal traits and growth backgrounds shape their self-efficacy through learning experiences, which in turn influences career decisions by shaping outcome expectations and interest development (Jiao et al., 2016; Lent & Brown, 2013). The social cognitive model places significant emphasis on adaptive career behaviors, which have been shown to facilitate individuals’ preparation for career changes and transitions. That is to say, an individual’s career adaptability has been demonstrated to enhance career competence, thereby increasing self-efficacy (Lent & Brown, 2013). Academic self-efficacy is defined as the expression of self-efficacy in learning activities—referring to an individual’s perceived capabilities to learn or perform actions at designated levels within academic contexts—which significantly impacts students’ learning interests and motivation and serves as a robust predictor of academic performance (Schunk & DiBenedetto, 2022). Longitudinal studies have demonstrated that self-efficacy pertaining to undergraduate academic skills and behaviors significantly predicts students’ recent academic performance and enhances their long-term learning experiences (Akomolafe et al., 2013; Ferla et al., 2009; McWhirter et al., 2000). Nevertheless—despite academic self-efficacy being a pivotal psychological construct that influences students’ academic performance and career decision making (Müller, 2014; Robertson, 2013)—there is a paucity of empirical studies examining the relationship between career education participation and academic self-efficacy, as well as its underlying mechanisms. This variable was selected as the outcome in order to further enhance our understanding of academic self-efficacy and the mechanisms through which career education affects academic performance.

### 1.1. Career Education Participation and Academic Self-Efficacy

Students’ engagement in career education fosters an enhanced understanding of self and society, thereby facilitating the formulation of career decisions that align with their unique characteristics. It stands to reason that such participation affected students’ self-efficacy positively. Despite the limited research on the impact of senior high school career education participation on academic self-efficacy, a number of other studies have also contributed to our understanding of the relationship between the two. A study involving American high school students found that, after a nine-week career-planning course intervention, the experimental group exhibited significantly higher vocational self-efficacy skills compared to the control group (McWhirter et al., 2000). Similarly, a study conducted in South Carolina found that targeted career counseling services increased students’ academic motivation, interest in learning, and readiness for college and work, thereby enhancing both their vocational and academic self-efficacy (Stipanovic et al., 2017). Other studies have yielded consistent findings (Ahn & Yu, 2020; Mahmud et al., 2022; Rabie et al., 2021). Therefore, the research proposes the following hypothesis: participation in career education can significantly enhance senior high school students’ academic self-efficacy.

### 1.2. The Mediating Role of Career Adaptability

Career adaptability is a psycho-social construct encompassing both the readiness and resources necessary for individuals to successfully navigate career tasks, occupational transitions, and unexpected challenges (Johnston et al., 2016). It is considered to be a crucial aspect of competency in individual career development, involving career curiosity, career concern, career control, and career confidence (Savickas & Porfeli, 2012). Career adaptability can prompt individuals to actively cope with challenges and is a necessary resource for successful career development (Chan & Mai, 2015). It has been demonstrated that participating in career-related activities can significantly promote a student’s level of career adaptability (Rabie et al., 2021). For instance, a randomized experiment conducted in the Czech Republic showed that participating in a 13-week career-planning course—with a focus on describing personal and vocational characteristics, developing career plans, enhancing job seeking ability, and developing information-searching abilities—could significantly improve career adaptability, collaboration skills, and career maturity among college students (Motlova & Honsova, 2021). Welde et al. (2015) found that engaging in self-exploration and career research activities could be beneficial for high school students. Such activities afford students an opportunity to reflect on their past, present, and future experiences, as well as engage in career-planning processes that are relevant to their future aspirations. The provision of career education activities by educational institutions represents the principal method by which senior high school students may gain insight into potential career pathways. In light of the aforementioned research findings, it can be posited that engagement in such activities may facilitate the cultivation of senior high school students’ career adaptability.

Existing research has indicated that the promotion of career adaptability could benefit the development of adolescents in many aspects, such as occupational self-efficacy, adapting responses, life satisfaction, and self-rated health (Hirschi et al., 2015; Johnston et al., 2016). Although there is a paucity of empirical evidence substantiating the assertion that career adaptability impacts academic self-efficacy, findings from research on the relationship between career adaptability and self-efficacy lend support to this proposition (Santilli et al., 2020). Moreover, there are notable correlations between career adaptability and self-efficacy of career decision-making (Rudolph et al., 2017), as well as between career adaptability and entrepreneurial self-efficacy (Minhui & Lingfeng, 2017). Since academic self-efficacy is a manifestation of self-efficacy within the academic domain, its relationship with career adaptability may be analogous to that observed in other types of self-efficacy.

Therefore, the current research posits that career adaptability may mediate the relationship between participation in career education and academic self-efficacy.

### 1.3. The Mediating Role of Learning Engagement

Learning engagement refers to the degree to which learners are prepared to invest in the learning process (Pike et al., 2011), reflecting the learners’ level of participation, concentration, and emotional state that they bring to the learning environment (Schaufeli, 2017). Learning engagement is composed of four dimensions, including vigor, dedication, and absorption. According to the social cognitive career theory, learning experiences are a crucial source of self-efficacy. A related study examining the relationship between cognitive engagement and self-efficacy found a significant correlation between high school students’ cognitive engagement and their academic self-efficacy (Ugwu et al., 2013). Additionally, emotional engagement, particularly absorption, is positively correlated with academic self-efficacy (Ocumpaugh et al., 2016).

On the other hand, studies have indicated a correlation between career education and levels of student engagement (Kenny et al., 2006; Orthner et al., 2013). Plasman (2018) employed data from the High School Longitudinal Study of 2009 to ascertain that the completion of students’ career plans is positively correlated with their engagement, as evidenced by two-level propensity score matching analyses. The aforementioned plans enable students to identify their long-term career goals and to ascertain what they expect to accomplish as they work toward their goals, thus encouraging students to be more closely involved in their studies. Furthermore, another study found that students who participated in career education programs exhibited the highest scores in career development skills, which are crucial for making career plans (Choi et al., 2015).

In light of the preceding discussions regarding participation in career education, learning engagement, and academic self-efficacy, the current research proposes that learning engagement functions as a mediator between career education participation and academic self-efficacy.

### 1.4. The Sequential Mediating Role of Career Adaptability and Learning Engagement

In addition to the assumption of independent mediating roles, career adaptability and learning engagement may also serve as sequential mediators between career education participation and academic self-efficacy, as evidenced by relative studies. According to Bandura (1982), self-efficacy is derived from four primary sources: successful experience, verbal persuasion, examples of the success of others, and physiological conditions. Demonstrating to senior high school students the accomplishments of their peers is a pivotal educational activity in fostering career resilience. Consequently, we contend that engagement in career education activities is a significant source of self-efficacy for student learning. Despite the absence of direct evidence regarding the relationship among senior high school students, previous studies conducted on adults have demonstrated that career adaptability has a positive impact on an individual’s academic performance (Chen & Zhang, 2023; Guo et al., 2014; Yu & Li, 2015). Yu and Li (2015) identified that students exhibiting elevated levels of career adaptability exhibited robust motivation to learn, a tendency to proactively establish learning objectives, and an ability to effectively plan coursework and personal development. Moreover, research conducted by Chen and Zhang (2023) yielded analogous results, indicating that career adaptability positively influences the learning engagement of Chinese vocational nursing students. A study of Chinese adolescents demonstrated a positive correlation between four aspects of career development (career feeling, career belief, career exploration, and career planning) and adolescents’ academic self-efficacy. Those showing high career exploration and planning showed significantly higher levels of academic self-efficacy (Deng et al., 2021). One potential explanation can be derived from career construction theory. Career construction theory posits that career adaptability is a self-regulatory capacity which enables individuals to continuously adjust their responses to the environment (Savickas, 2002). This theory suggests that individuals with high levels of career adaptability possess greater mental resources and enhanced adaptability. They are adept at anticipating future trends and proactively preparing to meet learning challenges, thereby effectively adapting to changes in their career environment (Chen & Zhang, 2023). Another study on career education showed that students who completed the career development course had a higher level of career decision-making self-efficacy (Reese & Miller, 2006).

It is evident that career education participation constitutes a pivotal method for cultivating career adaptability among senior high school students. The present study proposes a hypothesis that career adaptability and learning engagement function as sequential mediators in the relationship between career education participation and academic self-efficacy.

### 1.5. The Current Study

**Figure 1:**
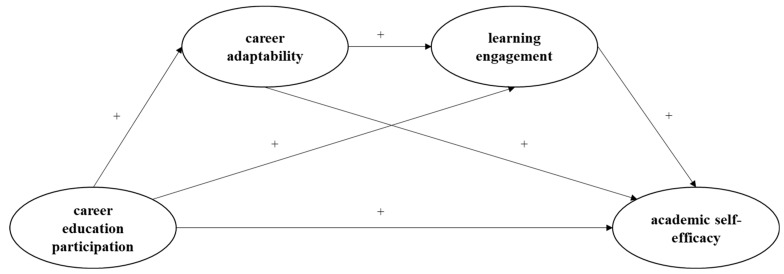
The proposed model of the current study.

The current study aims to examine the relationship between career education and academic self-efficacy among Chinese senior high school students, as well as indicate the independent and sequential mediating effects of career adaptability and learning engagement in the relationship (Figure 1). Four hypotheses were derived based on our review of the existing literature.

**Hypothesis** **1.** *Participation in career education is positively associated with academic self-efficacy.*

**Hypothesis** **2.** *Career adaptability plays a mediating role in the relationship between career education participation and academic self-efficacy.*

**Hypothesis** **3.** *Learning engagement mediates the association between career education participation and academic self-efficacy.*

**Hypothesis** **4.** *Career adaptability and learning engagement sequentially mediate the relationship between career education participation and academic self-efficacy.*

## 2. Methods

### 2.1. Participants

A total of 848 senior high school students (male = 390, female = 458, *M_age_* = 16.88, *SD_age_* = 3.56) were recruited to participate in the research. Of the total number of participants, 251 were in tenth grade, 515 were in eleventh grade, and 82 were in twelfth grade. The sample included 406 students from urban areas and 442 from rural areas.

### 2.2. Measures

#### 2.2.1. Career Education Participation

The Career Education Participation Questionnaire is a four-item instrument developed with reference to prior practice (Cui et al., 2022) and based on the key learning content of career education in Chinese senior high schools. The items encompass four categories of career exploration: career awakening, self-awareness, career exploration, and career decision making and management. These are specific to the educational context (e.g., “How many times have you participated in self-awareness classes or activities (such as learning about your personality, strengths, hobbies, etc.) in senior high school?”; “How many times did you participate in career exploration classes or activities (learning about college entrance exam policies, college majors, social careers, etc.) in senior high school?”). In the questionnaire, participants are asked to report on a 5-point scale ranging from 1 (*seldom*) to 5 (*very often*). Higher average scores indicate a higher level of career education participation. The Cronbach’s alpha for this study was 0.92.

#### 2.2.2. Career Adaptability

Career adaptability was measured using the 24-item Career Adaptability Scale developed by Hou et al. (2012) among Chinese senior high school students. These items involve age-related career behaviors (e.g., “I’m concerned about my career”; “I can make decisions on my own”; “I’m always curious about new opportunities”) that require participants to assess their level of competence to perform these behaviors on a 5-point scale ranging from 1 (*not strong*) to 5 (*extremely strong*). A higher average score indicates higher levels of career adaptability. The Cronbach’s alpha for this study was 0.96.

#### 2.2.3. Learning Engagement

Learning engagement was assessed using the 17-item Learning Engagement Scale developed by Fang et al. (2008), which was constructed with three dimensions: vigor, dedication, and absorption. These items inquire about the frequency with which participants engage in certain learning behaviors (e.g., “I feel energetic when I’m studying”; “I feel happy when I’m fully engaged in my studies”; “I find learning challenging”). Participants were required to self-report on a 7-point scale ranging from 1 (*never*) to 7 (*always*). Higher average scores indicate higher levels of learning engagement. The Cronbach’s alpha for this study was 0.96.

#### 2.2.4. Academic Self-Efficacy

Academic self-efficacy was assessed using the 22-item Academic Self-Efficacy Questionnaire developed by Liang (2000). This was constructed with two dimensions: academic ability self-efficacy and academic behavior self-efficacy. Participants were required to self-report the extent to which their own learning behaviors matched those mentioned in the questionnaire (e.g., “I believe I have the ability to do well in study”; “I like to choose challenging learning tasks”; “I always underline key sections in my books or notebooks to help with learning”) on a 5-point scale ranging from 1 (*totally mismatched*) to 5 (*totally matched*). Higher average scores indicate higher levels of academic self-efficacy. The Cronbach’s alpha for this study was 0.90.

### 2.3. Procedure

With the approval of each school’s principal, class counselors explained the purpose of the research to students’ parents and obtained their consent. The trained class counselors informed the students about the purpose and requirements of the survey during class, emphasizing the anonymity of the survey and its independence from academic performance. The survey was then distributed and collected through an online questionnaire platform. Participants were informed that they needed to complete the questionnaire independently and had the right to withdraw from the survey at any time.

### 2.4. Data Analysis

Descriptive statistics and variables correlations were calculated using the SPSS 20.0, while the sequential mediation model was estimated by using Mplus 8.0. Gender and age were incorporated as covariates into the model. Bootstrapping—a statistical technique enabling the construction of confidence intervals and the execution of tests without reliance on the normality assumption inherent in traditional methods (Erceghurn & Mirosevich, 2008)—was employed to derive the estimators. The statistical significance of the paths and indirect effects was assessed using 5000 bootstrap samples. The model fit was assessed using the Tucker–Lewis index (*TLI*), the comparative fit index (*CFI*), the root mean square error of approximation (*RMSEA*), and the standardized root mean square residual (*SRMR*). An acceptable model fit was indicated by *TLI* ≥ 0.95, *CFI* ≥ 0.95, *RMSEA* ≤ 0.06, and *SRMR* ≤ 0.08 (Hu & Bentler, 1999).

## 3. Results

### 3.1. Common Method Bias Test

Harman’s one-factor test was adopted to assess the potential presence of common method bias (Zhou & Long, 2004). The analysis revealed that nine eigenvalues were above 1, with the first principal component explaining 37.40% of the variance; this was below the critical threshold of 40%, indicating that common method bias in the current study was not substantial.

### 3.2. Descriptive Statistics and Correlations

The results of the descriptive analysis and correlation tests are summarized in Table 1. Significant associations were found between career education participation and career adaptability (*r* = 0.31, *p* < 0.001), learning engagement (*r* = 0.34, *p* < 0.001), and academic self-efficacy (*r* = 0.34, *p* < 0.001). Additionally, career adaptability was positively correlated with both learning engagement (*r* = 0.59, *p* < 0.001) and academic self-efficacy (*r* = 0.63, *p* < 0.001). Furthermore, learning engagement was found to be significantly related to academic self-efficacy (*r* = 0.72, *p* < 0.001). These significant relationships among the primary variables provide empirical support for the proposed sequential mediation model.

### 3.3. Sequential Mediation Test

**Figure 2:**
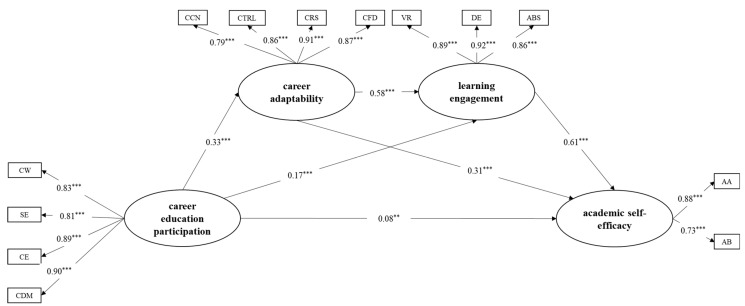
Result of sequential model test. The latent variable of career education participation was indicated by participation in classes or activities of career awakening (CW), self-awareness (SE), career exploration (CE), and career decision making and management (CDM). The latent variable of career adaptability was indicated by career control (CTRL), career concern (CCN), career curiosity (CRS), and career confidence (CFD). The latent variable of career adaptability was indicated by vigor (VR), dedication (DE), and absorption (ABS). The latent variable of career adaptability was indicated by academic ability self-efficacy (AA) and academic behavior self-efficacy (AB). The model was constructed with the inclusion of age and gender. For the sake of simplicity in the model, this is not illustrated in the figure. ** *p* < 0.01, *** *p* < 0.001.

The sequential mediation model was analyzed by using Mplus 8.0, and it demonstrated good fit indices: *RMSEA* = 0.058, *CFI* = 0.972, *TLI* = 0.965, *SRMR* = 0.031. The results show that the relationship between career education participation and academic self-efficacy was sequentially mediated by career adaptability and learning engagement (Figure 2). Additionally, career adaptability and learning engagement independently mediated the relationship between career education participation and academic self-efficacy.

We further analyzed the indirect effects. Bootstrapping analyses showed that the indirect effect of career education participation on academic self-efficacy through both career adaptability and learning engagement was significantly positive (*b* = 0.088, *p* < 0.001, 95% CI [0.067, 0.116]). Additionally, the indirect effect of career education participation on academic self-efficacy via career adaptability was significant and positive (*b* = 0.078, *p* < 0.001, 95% CI [0.050, 0.114]), as was the indirect effect via learning engagement (*b* = 0.080, *p* < 0.001, 95% CI [0.044, 0.121]). The results are presented in Table 2.

## 4. Discussion

The present study was designed to investigate the relationship between senior high school students’ participation in career education and their academic self-efficacy, as well as to explore the underlying mediating processes. This study, conducted with a sample of 848 senior high school students in China, revealed that participation in career education significantly enhances academic self-efficacy. Specifically, career adaptability and learning engagement act as sequential mediators in the relationship between career education participation and academic self-efficacy. These results underscore the substantial impact that career education activities in senior high schools can have on psychological factors that are integral to academic success.

### 4.1. The Relationship Between Career Education Participation and Academic Self-Efficacy

The findings confirm the initial hypothesis by revealing a significant positive correlation between students’ involvement in senior high school career education and their academic self-efficacy. In other words, those who participate more actively in career education tend to exhibit higher levels of academic self-efficacy, corroborating the insights from prior research on self-efficacy (Rabie et al., 2021). Career education plays a pivotal role in enhancing students’ self-awareness, their understanding of the external environment, and their ability to regulate their own learning processes. Furthermore, the establishment of career goals has a significant impact on students’ beliefs and behaviors, influencing their academic pursuits and personal development. When students articulate clear career development goals, they are more likely to demonstrate enhanced academic motivation, a heightened commitment to their studies, a propensity for undertaking more rigorous academic programs, and a bolstered belief in their capacity to overcome scholastic obstacles. Career education plays a pivotal role in fostering students’ comprehensive comprehension of their prospective professional paths, equipping them with a robust sense of direction. This directional clarity not only aids in charting a focused academic trajectory but also significantly boosts their academic self-efficacy (Stipanovic et al., 2017).

### 4.2. The Mediating Effect of Career Adaptability

The current findings indicate that engagement in career-oriented learning activities during senior high school significantly enhances students’ academic self-efficacy by fostering their career adaptability. Our research demonstrates that career adaptability functions as a pivotal mediator in the relationship between engagement in career education and academic self-efficacy, thereby enriching existing theoretical models. Previous studies have predominantly concentrated on the influence of career adaptability on decision making self-efficacy, vocational self-efficacy, and entrepreneurial self-efficacy (Hirschi et al., 2015; Zhang et al., 2024); meanwhile, they have failed to investigate the impact on academic self-efficacy and the mediating role of career adaptability in the context of career education engagement. This study addresses a significant gap in the existing literature. The findings demonstrate that students can enhance their career adaptability through the development of key competencies, including career concern, career control, and career confidence. These competencies empower students to forge meaningful connections with their societal context, actively seek out career-related information, and systematically acquire the knowledge and skills required to realize their career aspirations. This systematic approach not only boosts their self-efficacy but also paves the way for successful career progression (Santilli et al., 2020).

### 4.3. The Mediating Effect of Learning Engagement

This study demonstrated that learning engagement serves as a pivotal mediator in the relationship between students’ participation in career education and their academic self-efficacy. This indicates that participation in career education programs enhances students’ academic self-efficacy by positively influencing their level of learning engagement. This lends support to the proposition put forth by the SCCT that learning experiences play a pivotal role in shaping self-efficacy. One potential explanation for the mediating mechanism may be that participation in career education helps students recognize the relevance of their current studies to their future career paths, which in turn elevates their engagement in learning activities, thereby enhancing their confidence and competence in addressing academic challenges (Welde et al., 2015).

### 4.4. The Sequential Mediating Effect of Career Adaptability and Learning Engagement

The findings indicate that, in addition to the independent mediating roles of career adaptability and learning engagement, there is a notable chain mediation effect. Specifically, career adaptability and learning engagement function as sequential mediators in the relationship between participation in career education and academic self-efficacy. This discovery offers novel insights into the underlying mechanisms by which engagement in career education can enhance academic self-efficacy. Prior studies have predominantly concentrated on either the impact of career education participation on students’ career adaptability or on the influence of career adaptability on learning engagement and academic performance (Handoyo & Sulistiani, 2017; Motlova & Honsova, 2021). In contrast, the present study considers career education participation as the independent variable and academic self-efficacy as the dependent variable, with particular emphasis on the mediating roles of both career adaptability and learning engagement. This methodological approach has enabled us to gain a more nuanced and comprehensive understanding of the relationship under investigation. As previously stated, career education is a specialized form of instruction designed to cultivate students’ capacity to adapt to diverse career pathways. As students enhance their career adaptability, they tend to develop heightened motivation and interest in learning, as well as resilience when facing challenges (Chen & Zhang, 2023). This, in turn, promotes more profound engagement in academic pursuits. As they accumulate rich learning experiences over time, their academic self-efficacy is progressively bolstered. This process represents one of the key mechanisms by which participation in career education influences academic self-efficacy.

## 5. Limitations

This study confirmed that engaging in career education significantly enhances academic self-efficacy among a sample of Chinese senior high school students. It further investigated the underlying mechanisms through which career adaptability and learning engagement contribute to this positive effect. The results corroborate the notion that career education can positively influence students’ academic progress and provide empirical evidence supporting the practicality and efficacy of career education in the Chinese educational context. However, certain limitations of this study necessitate attention in future research endeavors.

Firstly, the cross-sectional nature of this study limits our ability to establish causal relationships. To better understand the dynamic interactions and causal links between the variables, subsequent studies should employ a longitudinal research design or experimental design. Secondly, the data were collected through self-reporting by the participants, which may introduce a level of bias due to subjective perceptions. Future studies could consider utilizing multiple data sources or objective measures to enhance the reliability and objectivity of the findings. Thirdly, academic self-efficacy, a psychological construct proven to significantly influence adolescent academic progress, was utilized as a proxy for academic development. While, in the Chinese educational landscape, academic achievement remains the primary benchmark recognized by parents and educators, academic self-efficacy has emerged as a crucial predictor of future academic success. Research has shown that early academic self-efficacy is positively correlated with higher academic performance in the later teenage years. Nonetheless, delving into the relationship between participation in career education and academic achievement can enhance our understanding of how career education impacts the academic development of adolescents, providing a more nuanced perspective on its role.

## 6. Conclusions

In conclusion, this research has demonstrated that participation in career-focused educational programs has a significant impact on academic self-efficacy. The study findings indicate that career adaptability and learning engagement are pivotal mediating factors, exerting direct and sequential mediating effects. These insights have significant implications for the design of educational interventions. The overarching objective of career education in senior high schools worldwide has been to empower students to achieve both academic excellence and career readiness. The findings of this investigation confirm that career education is an invaluable component of the educational landscape, significantly enhancing students’ academic self-efficacy. It is imperative that governments, especially in developing nations, create more favorable conditions for schools to integrate career education into their curricula. By doing so, they can foster a more engaged student body that actively participates in career education activities. This engagement will, in turn, establish a robust psychological base conducive to academic excellence.

## Figures and Tables

**Table 1:** Results of descriptive statistics and correlations (N = 848).

	*M (SD)*	1	2	3	4
1. Career education participation	2.27 (0.85)	1			
2. Career adaptability	4.01 (0.60)	0.31 ***	1		
3. Learning engagement	4.84 (1.04)	0.34 ***	0.59 ***	1	
4. Academic self-efficacy	3.50 (0.50)	0.34 ***	0.63 ***	0.72 ***	1

Note: *** *p* < 0.001.

**Table 2:** Results of indirect effects of the sequential mediation model (N = 848).

Pathways	*b*	95% CI	
		LL	UL
Total Effect	0.302 ***	0.233	0.370
Direct Effect CEP→ASE Indirect Effect	0.056 **	0.015	0.097
Total Indirect Effect	0.246 ***	0.196	0.299
CEP→CA→ASE	0.078 ***	0.050	0.114
CEP→LE→ASE	0.080 ***	0.044	0.121
CEP→CA→LE→ASE	0.088 ***	0.067	0.116

Note: CI = confidence interval; LL = lower limit; UL = upper limit; CEP = career education participation; CA = career adaptability; LE = learning engagement; ASE = academic self-efficacy. ** *p* < 0.01, *** *p* < 0.001.

## Data Availability

The datasets processed and analyzed during the current study are available from the corresponding/first author upon reasonable request.
